# Differential diagnostic importance of swept-source optical coherence tomography in ocular surface lesions: Swept-source OCT in ocular surface lesions

**DOI:** 10.1186/s12886-025-04137-1

**Published:** 2025-05-22

**Authors:** Kincső Kozma, Béla Kajtár, Zsuzsanna Zita Orosz, Bence Nagy, Adrienne Csutak, Eszter Szalai

**Affiliations:** 1 Department of Ophthalmology, University of Pécs Medical School, Rakoczi u. 2, Pecs, 7623 Hungary; 2 Department of Pathology, University of Pécs Medical School, Rakoczi u. 2, Pecs, 7623 Hungary; 3 Department of Ophthalmology, Albert Szent-Györgyi Medical Faculty, University of Szeged, Szeged, Hungary; 4 Department of Pathology, University of Szeged, Szeged, Hungary

**Keywords:** Conjunctiva, Cornea, Histopathology, Ocular surface, OSSN, OCT

## Abstract

**Background:**

The purpose of this retrospective study was to analyze the features of different ocular surface lesions using high resolution swept-source optical coherence tomography (OCT) and to correlate those characteristics with histopathologic alterations.

**Methods:**

Thirty-eight eyes of 37 patients (19 males and 18 females) were included in this study with a mean age of 60.36 ± 17.29 years years. Anterior segment imaging was performed with a swept-light source OCT system (ANTERION, Heidelberg Engineering, Germany) using the Imaging App. Clinical diagnosis based on the slit-lamp findings was compared with the OCT features of the lesion and the histopathology result.

**Results:**

Based on the OCT features, 11 lesions were in the epithelium, of which 5 had only epithelial component. Six growths had both epithelial and subepithelial components and 27 lesions were confined to the substantia propria. The OCT finding and histopathology result correlated in 57% (6/11) for epithelial involvement and in 84% (28/33) for subepithelial involvement. In 25 cases (65%), the clinical and histopathology diagnosis agreed. In 13 cases (35%), the clinical suspicion was different from the final histopathology diagnosis. Out of those 13 cases, high-resolution OCT findings were suggestive of the histopathology results in 8 cases.

**Conclusions:**

High-resolution swept-source OCT provided valuable information of the structure, topographic location and extent of an ocular surface lesion and was helpful in assisting the diagnosis.

## Background

Assessment of the ocular surface functional unit is an important part of the routine ophthalmological examination, which has traditionally been performed with slit-lamp. Corneal and conjunctival growths can be diagnosed with incisional or excisional biopsy. The recently introduced swept light source Fourier domain anterior segment optical coherence tomography (OCT) is based on low coherence interferometry [1]. Swept-source OCT devices can achieve a few-micron resolution; thus, this non-invasive technique is also referred to as optical biopsy [2–4]. A swept laser source operating at 1300 nm wavelength can achieve an axial resolution of ≤ 10 microns and a lateral resolution of 30 microns [5]. With this recent enhancement in resolution, ocular surface pathologies could be examined in tissue level [6–9]. Higher resolution enables clinicians to discriminate between epithelial and stromal lesions [8, 10].

Ocular surface lesions have a wide range of differential diagnosis, that includes degenerations, inflammations, benign and malignant melanocytic and non-melanocytic tumors. However, apart from epithelial and stromal conjunctival growths, tumors can be originated from the underlying structures and extend to the ocular surface. Diagnosis based on clinical presentation could be challenging due to the variable slit-lamp appearance of the lesions. The purpose of this retrospective study was to analyze the features of different typical and atypical ocular surface masses using high resolution swept-source OCT and to correlate those characteristics with histopathologic alterations.

## Methods

Anterior segment imaging was performed with a high resolution swept-light source Fourier-domain OCT system (ANTERION, Heidelberg Engineering, Heidelberg, Germany) using a 1310 nm wavelength laser source. Measurements were taken using the Imaging App of the anterior segment OCT and two scan types (linear and radial) were used centered at the corneal or conjunctival lesion.

Incisional or excisional biopsy was indicated in all cases and the sample was sent for histopathology evaluation. Tissue samples were stained with hematoxylin and eosin staining (H&E), Ki-67 and p40 markers were used for immunohistochemical (IHC) analysis.

Clinical diagnosis based on the slit-lamp findings was compared with the OCT features of the lesion and the histopathology result. In all cases, slit-lamp examinations and photographic documentation were performed and analyzed prior to the review of OCT images to establish a preliminary clinical diagnosis. Optical coherence tomography (OCT) evaluation was subsequently conducted independently, without access to histopathological findings. Histopathological analysis was performed last. This stepwise assessment enabled an unbiased evaluation of the diagnostic accuracy associated with each modality.

The retrospective study was performed in accordance with the tenets of the Helsinki Declaration and the protocol was approved by the University of Pecs Institutional Ethical Review Board (Number: KK/119-1/2020).

## Results

Thirty-eight eyes of 37 patients (19 males and 18 females) were included in this retrospective study. The mean age was 60.36 ± 17.29 years (ranging from 8 to 85 years). Demographic characteristics are shown in Table 1. Twenty-seven patients had excisional and 10 patients had incisional biopsy.

**Table 1:** Demographic characteristics of the study patients, clinical diagnosis, optical coherence tomography features and histopathology diagnosis of the lesions

No	Age	Sex	Eye	Location	Pigmentation	Clinical diagnosis	Optical coherence tomography alterations	Histopathology diagnosis	Clincal vs. OCT Agreement	Clinical vs. Histo Agreement	OCT vs. Histo Agreement
1	14	F	R	Temporal paralimbal, bulbar conjunctiva	Pigmented	Conjunctival nevus	Subepithelial growth without cyst	Pigmented conjunctival nevus	YES	YES	YES
2	18	M	R	Temporal paralimbal, bulbar conjunctiva	Pigmented	Conjunctival nevus	Subepithelial growth with cyst	Compound conjunctival nevus	YES	YES	YES
3	8	F	L	Temporal paralimbal bulbar conjunctiva	Pigmented	Conjunctival nevus	Subepithelial growth without cyst	Compound conjunctival nevus	YES	YES	YES
4	42	M	L	Temporal, bulbar conjunctiva	Pigmented	Conjunctival nevus	Subepithelial growth without cyst	Conjunctival nevus	YES	YES	YES
5	38	F	L	Temporal, bulbar conjunctiva	Pigmented	Conjunctival nevus	Subepithelial growth without cyst	Compound conjunctival nevus	YES	YES	YES
6	41	M	L	Temporal, bulbar conjunctiva	Pigmented	Conjunctival nevus	Subepithelial growth without cyst	Compound conjunctival nevus	YES	YES	YES
7	33	F	R	Temporal, bulbar conjunctiva	Pigmented	Conjunctival nevus	Subepithelial growth without cyst	Conjunctival nevus	YES	YES	YES
8	36	F	L	Temporal bulbar conjunctiva	Pigmented	Conjunctival naevus	Subepithelial growth with cysts	Conjunctival naevus	YES	YES	YES
9	67	F	R	Temporal bulbar conjunctiva	Pigmented	Conjunctival nevus	Subepithelial growth with cysts	Conjunctival nevus	YES	YES	YES
10	24	M	R	Inferior forniceal conjunctiva	Pigmented	Conjunctival blue nevus	Thickened inhomogenous substantia propria with hyporeflective space	Venous lake	NO	NO	YES
11	75	M	R	Bulbar conjunctiva, in 360° & caruncle	Pigmented	PAM	Basal epithelial hyperreflective band Inhomogenous thickened subepithelium with vacuoles	PAM, without atypia	YES	YES	YES
12	54	F	L	Upper nasal bulbar conjunctiva	Pigmented	PAM	Basal epithelial hyperreflective band	PAM, without atypia	YES	YES	YES
13	71	M	L	Tarsal & bulbar conjunctiva, fornix & caruncle	Pigmented	PAM	Basal epithelial hyperreflective band thickened hyperreflective inhomogeneous subepithelial mass with cysts	Conjunctival melanoma arising in PAM with atypia	NO	NO	YES
14	41	M	R	Caruncle, nasal bulbar conjunctiva	Non-pigmented	Oncocytoma	Multiseptated cysts in the thickened stroma	Conjunctival naevus	NO	NO	YES
15	59	F	L	Nasal paralimbal, bulbar conjunctiva	Non-pigmented	Conjunctival cyst	Thickened epithelium, subepithelial homogenous hyporeflective mass	Squamous cell papilloma	NO	NO	NO
16	61	F	L	Nasal bulbar conjunctiva	Non-pigmented	Conjunctival cyst	Subepithelial, homogenous hyporeflective space	Conjunctival inclusion cyst	YES	YES	YES
17	47	M	R	Nasal, bulbar conjunctiva	Non-pigmented	Conjunctival cyst	Subepithelial, homogenous hyporeflective space	Conjunctival inclusion cyst	YES	YES	YES
18	45	M	L	Tarsal conjunctiva, upper eyelid	Non-pigmented	Conjunctival papilloma	Thickened hyperreflective epithelium, homogenous thickened hyporeflective subepithelium with finger-like projections	Hordeolum	NO	NO	NO
19	58	M	R	Caruncle, lower tarsal conjunctiva	Non-pigmented	Papilloma	Thickened hyperflective epithelium	Squamous papilloma	YES	YES	YES
20	54	F	L	Nasal bulbar conjunctiva	Non-pigmented	Pinguecula	Subepithelial inhomogenous hyperreflective growth	Pterygium	YES	NO	YES
21	43	M	L	Nasal bulbar conjunctiva, encroaching the cornea	Non-pigmented	Pterygium	Subepithelial inhomogenous hyperreflective growth	Pterygium	YES	YES	YES
22	50	F	R	Nasal bulbar conjunctiva, encroaching the cornea	Non-pigmented	Pterygium	Subepithelial inhomogenous hyperreflective growth, with fine hyperreflective lines	Pterygium	YES	YES	YES
23	64	M	L	Nasal bulbar conjunctiva, encroaching the cornea	Non-pigmented	Pterygium	Subepithelial inhomogenous hyperreflective growth	Pterygium	YES	YES	YES
24	81	F	R	Nasal bulbar conjunctiva, encroaching the cornea	Non- pigmented	Atypical pterygium	Thickened hyperreflective subepithelium with posterior shadowing	Pterygium	YES	YES	YES
25	66	M	L	Nasal, bulbar conjunctiva	Non-pigmented	Pterygium recurrence	Thickened hyperreflective epithelium, thickened subepithelial lamellar inhomogeneous mass with cysts	Epithelium with no dysplasia, chronic inflammation in stroma	NO	NO	YES
26	68	F	R	Nasal, inferior bulbar conjunctiva	Non-pigmented	Lymphangiectasia	Inhomogenous hyperreflective subepithelial mass, with dilated vascular channels and edema	Lymphangiectasia	YES	YES	YES
27	68	F	L	Superior, nasal, bulbar conjunctiva	Non-pigmented	Lymphoma	Thickened homogeneous hyporeflective subepithelial mass with hyperreflective fine lines and edema	Conjunctival MALT lymphoma	YES	YES	YES
28	75	F	L	Inferior, bulbar & forniceal conjunctiva	Non-pigmented	Lymphoma	Homogenous hyperreflective subepithelial mass	Conjunctival MALT lymphoma	YES	YES	YES
29	70	F	L	Upper bulbar conjunctiva	Non-pigmented	Lymphoma	Hmogenous hyperreflective subepithelial mass	Lymphoma	YES	YES	YES
30	73	M	L	Inferior conjunctiva, 180° around the limbus	Non-pigmented	Lymphoma	Thickened hyperreflective, epithelium, abrupt transition from normal to abnormal epithelium, epithelial cystic space representing cross section of a vessel	Squamous cell carcinoma	NO	NO	YES
31	72	M	R	Temporal, superior bulbar conjunctiva, reaching the cornea	Non-pigmented	OSSN	Thin hyperreflective epithelium, edematous hyporeflective substantia propria	Chronic conjunctivitis	NO	NO	NO
32	79	M	L	Temporal, paralimbal conjunctiva	Non-pigmented	OSSN	Thickened hyperreflective epithelium, inhomogenous subepithelial mass with vascular channels	Papillomatous epithelium	NO	NO	YES
33	85	F	R	Bulbar conjunctiva, in 360° & cornea	Non-pigmented	OSSN	Variable epithelial thickness, thickened inhomogenous subepithelial mass	Granulation tissue	NO	NO	NO
34	70	F	R	Inferior cornea	Non-pigmented	OSSN	Thin epithelium, subepithelial inhomogenous hyperreflective large mass	Salzmann nodular degeneration	NO	NO	NO
35	54	F	R	Infero-nasal, paracentral cornea	Non-pigmented	OSSN	Hyperreflective homogenous subepithelial mass with cL and shadowing	Malignant melanoma	NO	NO	YES
36	73	M	R	Limbal conjunctival 6–9 o’clock	Non-pigmented	Leukoplakic OSSN	Thickened epithelium	CIN with mild dysplasia	YES	YES	YES
37	72	M	R	Inferior cornea	Non-pigmented	OSSN	Homogenous, hyperreflective, extremely thickened epithelium	CorIN	YES	YES	YES
38	80	M	L	Temporal bulbar, upper tarsal conjunctiva, encroaching the cornea	Non-pigmented	OSSN	Homogenous, hyperreflective, extremely thickened epithelium	Squamous cell carcinoma in situ	YES	YES	YES

PAM = Primary acquired melanosis; OSSN = Ocular surface squamous neoplasia; MALT = Mucosa associated lymphoid tissue; CIN = Conjunctival intraepithelial neoplasia; CorIN = Corneal intraepithelial neoplasia

Thirteen lesions were melanotic and 25 lesions were non-pigmented. Based on the OCT features, 11 lesions were in the epithelium, of which 5 had only an epithelial component. Six growths had both epithelial and subepithelial components and 27 lesions were confined to the substantia propria. The OCT finding and the histopathology result correlated in 57% (6/11) for epithelial involvement and in 84% (28/33) for subepithelial involvement. In 25 cases (65%), the clinical and histopathology diagnosis agreed. In 13 cases (35%) (case No. 10, 13, 14, 15, 18, 20, 25, 30, 31, 32, 33, 34, 35), the clinical suspicion was different from the final histopathology diagnosis. Out of those 13 cases, in 8 cases (case No. 10, 13, 14, 20, 25, 30, 32, 35), the high-resolution OCT findings were suggestive of the histopathology results. In 5 cases (case No. 15, 18, 31, 33, 34), the OCT was not able to change our clinical suspicion based on the slit lamp appearance of the lesion.

Based on the histopathology reports, 7 tumors were malignant (2 malignant melanomas, 3 conjunctival lymphomas and 2 squamous cell carcinomas), 4 tumors were premalignant (2 PAM, 2 corneal intraepithelial neoplasia), 3 lesions were of inflammatory origin and the others were benign growths (10 conjunctival nevi, 3 squamous cell papillomas, 5 pterygia, 2 conjunctival inclusion cysts, 1 granulation tissue, 1 Salzmann nodular degeneration, 1 lymphangiectasia, 1 venous lake of the conjunctiva).

**Fig. 1:**
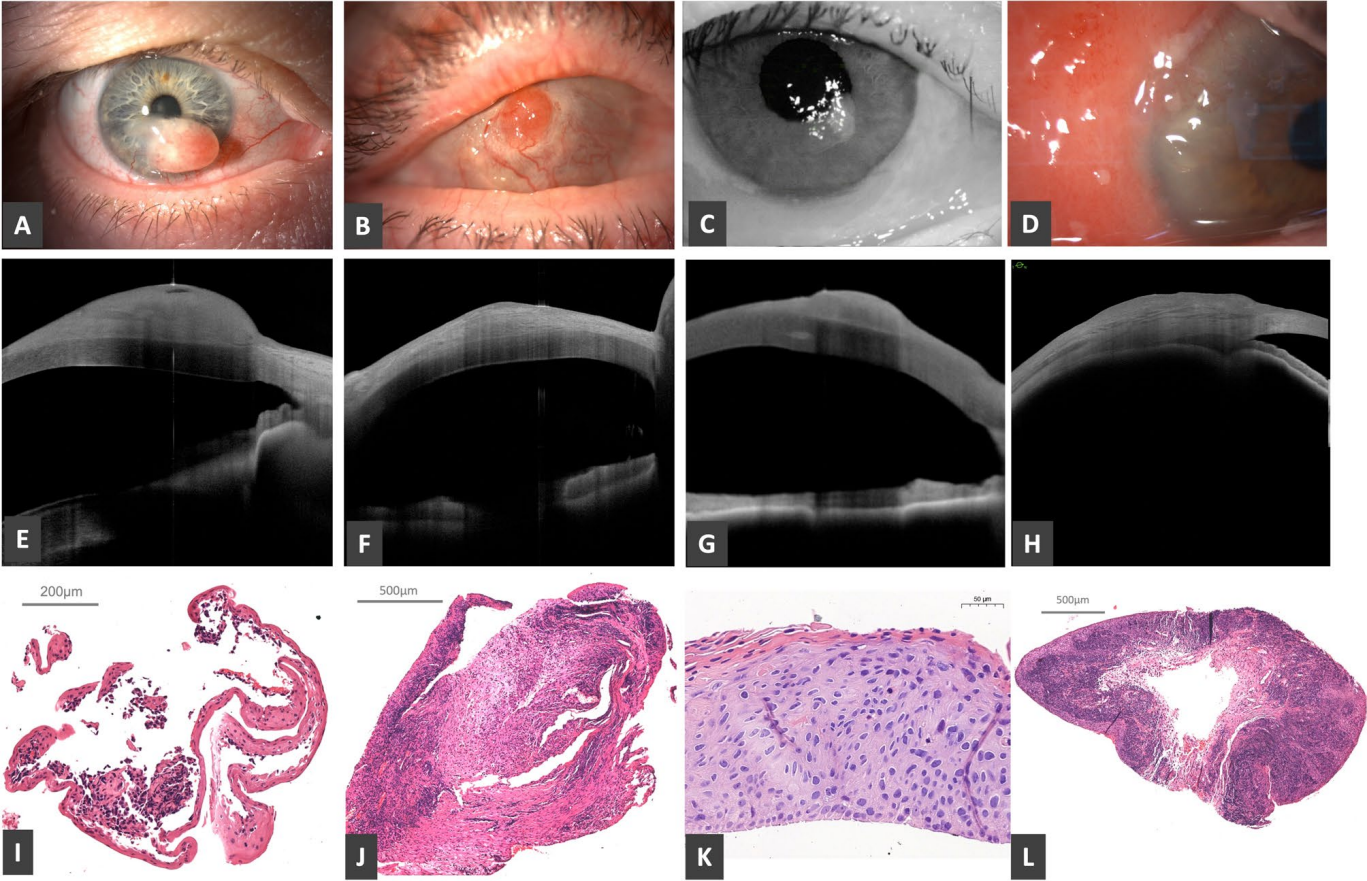
Slit-lamp photos **(A-D)**, anterior segment OCT appearance **(E-H)** and histopathology images **(I-L)** of different ocular surface lesions with corneal involvement. **A**,** E**,** I**: case No. 35, malignant melanoma. **B**,** F**,** J**: case No. 33, granulation tissue. **C**,** G**,** K**: case No. 37, corneal intraepithelial neoplasia. **D**,** H**,** L**: case No. 31, chronic conjunctival inflammation

Ten lesions showed corneal involvement. In Fig. 1, we present four cases in which the clinical diagnosis was ocular surface squamous neoplasia. However, the histopathological diagnosis matched the clinical diagnosis in only one case. In case no. 35, the AS-OCT image showed normal epithelium, a hyperreflective homogeneous subepithelial mass with clefts and shadowing, but, the histopathological result confirmed malignant melanoma. In this case, the clinical and histopathological diagnoses did not match; however, the OCT findings were suggestive of the histopathological diagnosis. In case no. 33, the OCT images supported our presumptive diagnosis, showing variable epithelial thickness and a thickened, inhomogeneous subepithelial mass. However, histopathology revealed granulation tissue. In case no. 37, the OCT findings showed a homogeneous, hyperreflective, and extremely thickened epithelium with a normal stroma. The histopathological diagnosis confirmed corneal intraepithelial neoplasia. Both the OCT findings and the histopathological results supported the clinical diagnosis. In case no. 31, the OCT images showed a thin, hyperreflective epithelium and an edematous, hyperreflective substantia propria. However, histopathology revealed chronic conjunctivitis. The clinical diagnosis did not match the OCT findings or the histopathological results, and the OCT findings were not specific to chronic conjunctivitis.

**Fig. 2:**
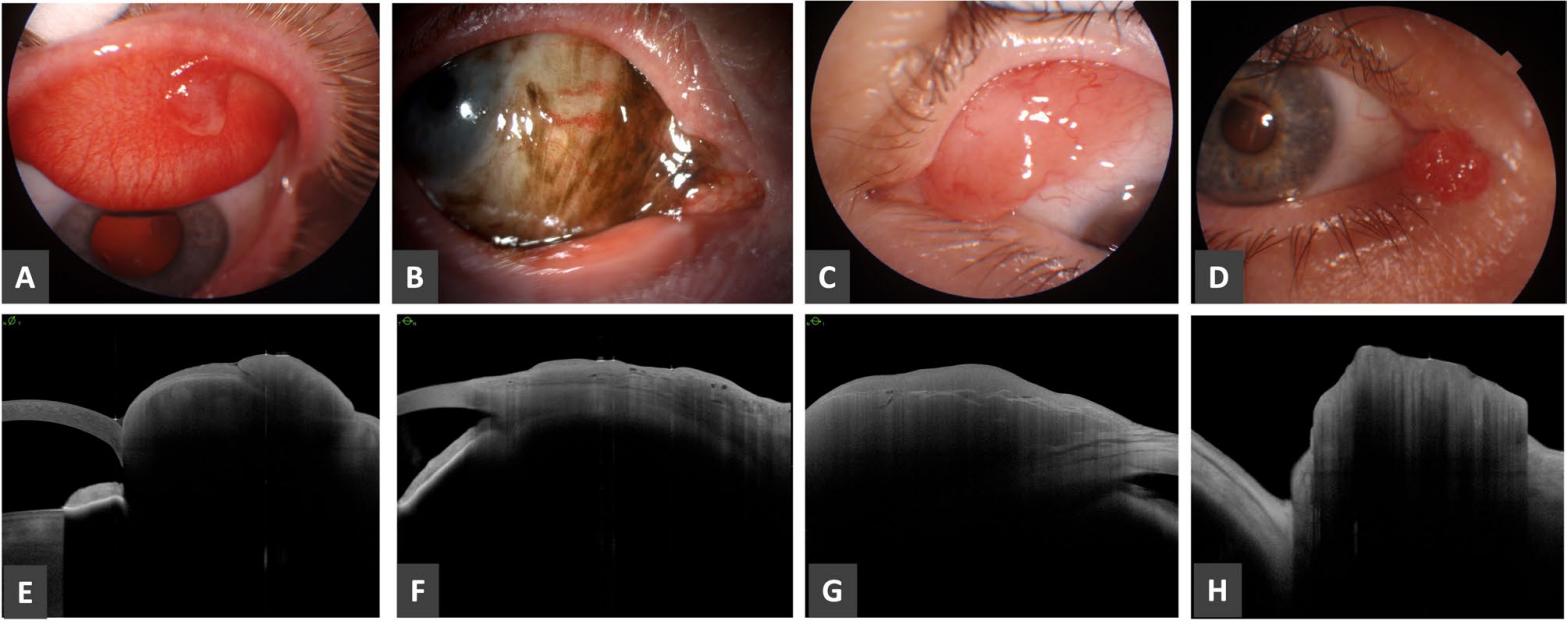
Slit-lamp **(A-D)** and anterior segment OCT appearance **(E-H)** of ocular surface lesions in different topographic locations. **A**,** E**: case No. 18, inflammatory mass on the upper tarsal conjunctiva. **B**,** F**: case No. 11, primary acquired melanosis involving the caruncle. **C**,** G**: case No. 27, lymphoma involving the superior bulbar conjunctiva. **D**,** H**: case No. 40, squamous papilloma originating from the caruncle

Three growths affected the forniceal conjunctiva, 3 masses were on the tarsal conjunctiva and 4 lesions reached the caruncle, Fig. 2 presents four of these cases. In case no. 18 the clinical diagnosis was conjunctival papilloma, while the OCT images presented thickened hyperreflective epithelium, homogenous thickened hyporeflective subepithelium with finger-like projections, but the histopathology result was hordeolum. In this case, the clinical diagnosis did not match either the OCT findings or the histopathological results, and the OCT findings were not specific to the final diagnosis. In case no. 11, the clinical diagnosis was Primary acquired melanosis, the OCT findings presented basal epithelial hyperreflective band, inhomogenous thickened subepithelium with vacuoles. The histopathological diagnosis confirmed the clinical suspicion, PAM without atypia. Lymphoma was the clinical diagnosis in case no. 27, which was confirmed by the histopathologycal result. On the OCT images, a thickened homogeneous hyporeflective subepithelial mass with hyperreflective fine lines and edema was notable, it correlated with the diagnosis. In case no. 19, the diagnosis was papilloma. In this case, the clinical diagnosis, OCT findings, and histopathological results were in agreement. The OCT findings showed a thickened, hyperreflective epithelium.

**Fig. 3:**
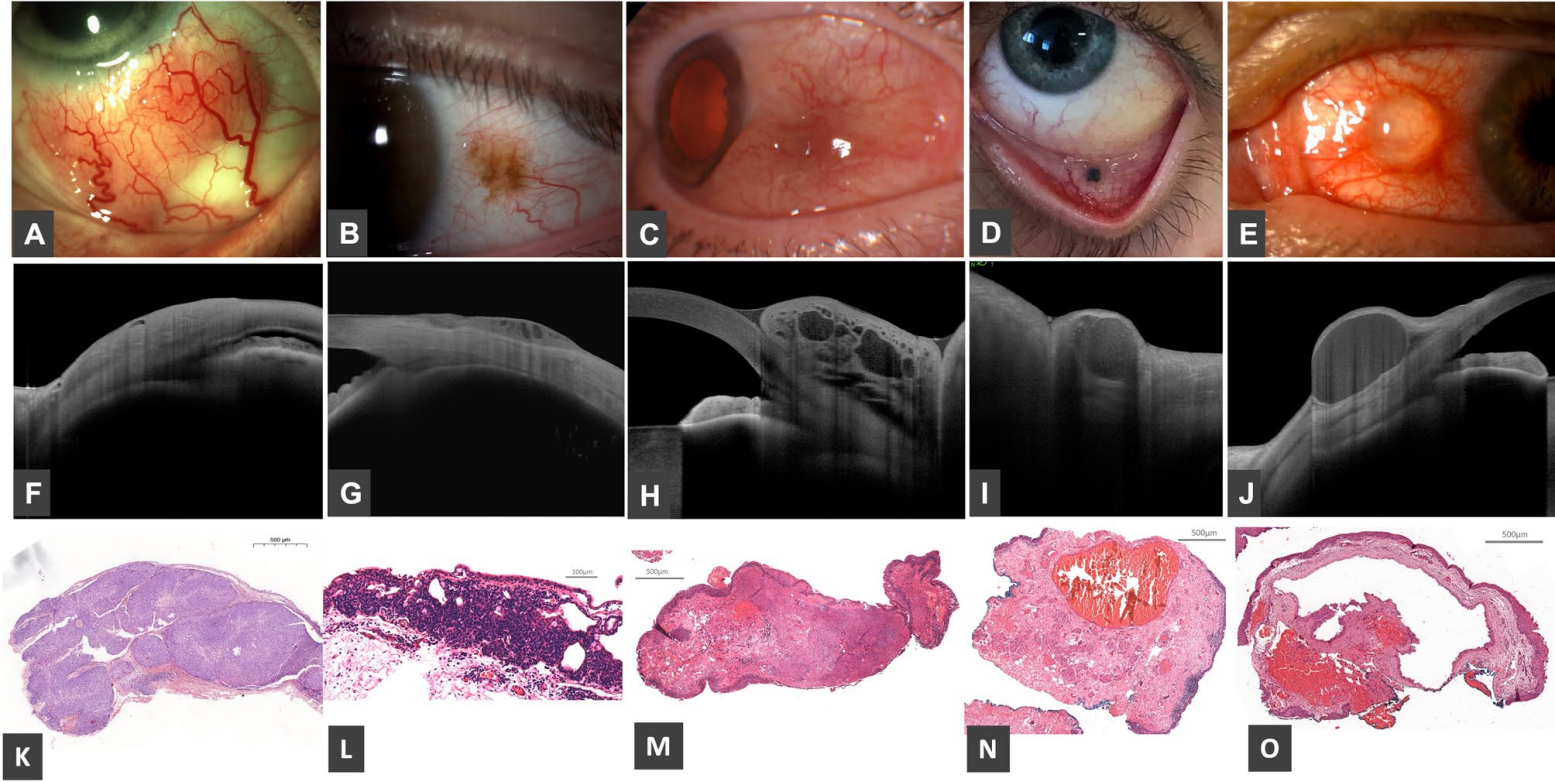
Slit-lamp photos **(A-E)**, anterior segment OCT appearance **(F-J)** and histopathology photos **(K-O)** of different ocular surface lesions with cystic spaces representing intralesional cysts and vascular channels. **A**,** F**,** K**: case No. 30, epithelial cystic space representing cross section of a feeder vessel in squamous cell carcinoma. **B**,** G**,** L**: case No. 2, intralesional cysts in compound nevus. **C**,** H**,** M**: case No. 26, dilated vascular channels in the substantia propria. **D**,** I**,** N**: case No. 10, cross section of a subepithelial dilated vessel (vein) in venous lake. **E**,** J**,** O**: case No. 16, inclusion cyst

Cystic spaces were identified in 13 cases with different etiologies. In eight cases, the cysts were intralesional, while in five cases, they were extralesional. Figure 3 shows five different cases with intralesional cysts. Case no. 30 epithelial cystic spaces represent cross section of feeder vessel. The clinical diagnosis was lymphoma, the OCT described thickened hyperreflective, epithelium, abrupt transition from normal to abnormal epithelium, epithelial cystic. However, the histopathology resulted in squamous cell carcinoma. On the OCT the abrupt transition from normal to abnormal epithelium suggested OSSN. Case no. 2 represents a conjunctival nevus with intralesional cystic spaces. Our clinical diagnosis aligned with the histopathological results, and the OCT findings—showing subepithelial growth with cysts—correlated with the diagnosis. Inhomogenous hyperreflective subepithelial mass, with dilated vascular channels and edema were described on the OCT in case no. 26. The clinical suspicion was lymphangiectasia, which correlated with the OCT finding and the histopathology. In case no. 10, the clinical diagnosis was conjunctival blue nevus. OCT revealed a thickened, inhomogeneous substantia propria with a hyporeflective space, representing the cross-section of a subepithelial dilated vessel (vein) in a venous lake, which was suggestive of the histopathological findings. The histopathological result was venous lake. Case no. 16 presents a conjunctival inclusion cyst, with the OCT showing a subepithelial, homogeneous, hyporeflective space. The clinical diagnosis, OCT findings, and histopathological results were in agreement.

Clinical and histopathological diagnoses did not align in six cases where both the epithelial and subepithelial layers were involved. This discrepancy was particularly notable in distinguishing bening and premalignant from malignant lesions. Among these cases, OCT findings were suggestive of the histopathological diagnosis in two instances.

A subgroup analysis was performed to evaluate pigmented and non-pigmented lesions separately. The pigmented subgroup included 13 patients (6 males and 7 females) with a mean age of 40.07 ± 21.65 years (range: 8–75 years). Histopathological examination revealed 10 benign, 2 premalignant and 1 malignant lesion, including 9 conjunctival nevi, 1 venous lake, 2 cases of primary acquired melanosis and 1 melanoma arising from PAM with atypia. Clinical and histopathological diagnoses were concordant in 11 cases, while in the remaining 2 cases, the OCT findings were consistent with the histopathological results.

In the non-pigmented subgroup, 25 patients were analyzed, comprising 13 males and 12 females, with a mean age of 64.52 ± 12.85 years (range: 41–85 years). Histopathological assessment identified 17 benign, 2 premalignant, and 6 malignant lesions. The distribution of diagnoses included 2 conjunctival cysts, 1 conjunctival nevus, 3 squamous papillomas, 5 cases of pterygium, 3 inflammatory lesions, 1 lymphangiectasia, 1 granulation tissue, 1 Salzmann’s nodular degeneration, 2 cases of conjunctival intraepithelial neoplasia, 2 squamous cell carcinomas, 3 lymphomas, and 1 amelanotic melanoma. Agreement between clinical and histopathological diagnoses was observed in 14 cases, while among the remaining 11 cases, OCT findings supported the histopathological diagnosis in 6 cases.

## Discussion

Swept source OCT is an advanced form of Fourier domain OCT that utilizes a wavelength-tunable laser source [1]. Compared to time-domain and spectral-domain OCT systems, swept source OCT offers several advantages [11]. In addition to its high axial and lateral resolution (≤ 10 microns and ≤ 30 microns, respectively), it enables deep tissue imaging with a 6 mm penetration depth and a broad 16 × 16 mm horizontal and vertical scan range. The 1310 nm wavelength laser allows for better absorption by water, enhancing the safety of the procedure (with less than 6% of the light energy reaching the retina) while also facilitating deeper scleral penetration and more detailed visualization of irido-corneal angle structures [11]. The purpose of this study was to show the role of high-resolution swept-source OCT in assisting the diagnosis of ocular surface lesions.

With recent advancements in technology, high-resolution OCT has been demonstrated to precisely identify OSSN with the following features: epithelial thickening and hyperreflectivity, abrupt transition zone between normal and abnormal tissue [12]. We observed similar OCT characteristics of our ocular surface lesions to those reported in the literature [4, 6–10, 12–19]. In squamous cell carcinoma, thickened epithelium, abrupt transition from normal to abnormal epithelium was observed using high-resolution OCT. In squamous papilloma, thickened hyperreflective epithelium was seen with normal substantia propria. In conjunctival nevus, normal epithelial thickness and subepithelial growth was found with or without intralesional cysts. In primary acquired melanosis without atypia normal epithelium thickness and hyperreflective basal epithelium were observed and in ocular surface malignant melanoma, basal epithelial hyperreflective band and thickened hyporeflective inhomogeneous subepithelial mass was observed with cysts or clefts. In our study, the anterior segment OCT was not able to differentiate between PAM and conjunctival/corneal malignant melanoma. In pterygium, normal epithelial thickness and subepithelial inhomogeneous hyperreflective growth was seen with or without fine hyperreflective lines. In conjunctival lymphoma, normal epithelium and thickened homogeneous hyporeflective subepithelial mass was documented with or without hyperreflective fine lines and stromal edema. As previously described, conjunctival epithelial layer could be visualized with anterior segment OCT only in cases of subepithelial nevi, while no distinct conjunctival epithelium could be detected in the junctional and compound types [20].

In our study, eleven lesions with different etiologies were identified as having cystic changes intralesionally. Based on high-resolution OCT, we were able to identify what the cystic spaces represented. In squamous cell carcinoma, cystic spaces in the thickened epithelium represented cross-sections of feeder vessels. Intralesional cysts were identified in compound nevus and PAM. Dilated vascular (lymphatic or vein) channels in the substantia propria were observed in lymphangiectasia and venous lake, respectively. In cases with inclusion cysts, the cystic cavity contained hyporeflective, homogenous proteinaceous material. Vempuluru et al. characterized two types of cysts on OCT: type 1 cysts were smaller in size, round to oval with hyporeflective contents corresponding to the blood vessels and type 2 were larger and irregular in shape containing degenerated cellular material [19]. According to Shields et al., cysts were noted in 70% of the compound nevi, 58% of the subepithelial nevi, 40% of the junctional nevi, and 0% of the blue nevi [21]. In papilloma, intrinsic spaces were thought to represent a cross-sectional view of the lumen of a vessel or protein-filled cysts [13]. Cases where both the epithelial and subepithelial layers were affected posed greater challenges in establishing an accurate diagnosis based on the OCT findings. This was particularly evident in distinguishing benign and premalignant lesions from malignant ones. These findings highlight the limitations of clinical assessment in detecting deeper structural involvement and emphasize the potential role of OCT in improving diagnostic accuracy by providing additional morphological information.

The subgroup analysis revealed distinct patterns in the diagnostic characteristics of pigmented and non-pigmented conjunctival lesions. In the pigmented subgroup, the majority of cases were benign, with conjunctival nevi being the most common diagnosis. Clinical and histopathological diagnoses showed a high concordance rate (11/13 cases, 84.6%), and in the remaining two cases, OCT findings were consistent with the histopathological results. In contrast, the non-pigmented subgroup demonstrated a more diverse histopathological profile, including a higher proportion of malignant cases (6/25, 24%) compared to the pigmented group (1/13, 7.7%). The clinical-histopathological agreement rate was lower (14/25 cases, 56%), but OCT findings contributed to aligning the diagnosis with histopathology in an additional 6 cases. These findings suggest that clinical diagnosis alone may be more reliable for pigmented lesions, whereas non-pigmented lesions pose greater diagnostic challenges. OCT appears to be a valuable adjunctive tool, particularly in cases where clinical and histopathological diagnoses initially differ. However, the significant difference in the number and diversity of cases between the two subgroups may have influenced the results, highlighting the need for further studies with a more balanced distribution of lesions.

In a previous study, authors found an excellent ability of anterior segment OCT for detection of epithelial involvement (sensitivity of 100% and specificity of 92%), and for subepithelial involvement was (sensitivity of 98% and specificity of 100%) [19]. We observed a 57% agreement for epithelial involvement and an 84% agreement for subepithelial involvement between the high-resolution OCT and histopathology. Atallah et al. concluded that the OCT devices they used cannot detect deep invasion of the tumor or grading [16]. Also, images of the inferior fornix and caruncle are not provided ideally with that device. However, three of our lesions (melanoma arising in PAM, venous lake and lymphoma) affected the fornix, 3 masses grew (squamous papilloma, hordeolum, squamous cell carcinoma) on the tarsal conjunctiva, four lesions (melanoma arising in PAM and PAM) reached the caruncle and the current swept source OCT provided high-resolution images of lesions in those special locations.

While AS-OCT has proven to be a valuable tool in evaluating ocular surface lesions, future studies could benefit from comparisons with other anterior segment imaging modalities, such as ultrasound biomicroscopy (UBM). UBM, which operates at a lower frequency (typically 35–50 MHz) than AS-OCT, provides deeper tissue penetration, making it particularly useful for assessing lesions that extend beyond the epithelial and subepithelial layers into deeper stromal or subconjunctival regions. In cases where AS-OCT findings suggest subepithelial involvement but lack sufficient depth resolution, UBM could help determine whether the lesion extends into deeper structures. This could be particularly relevant in distinguishing PAM with atypia from early invasive melanoma, as deeper invasion is a key factor in guiding clinical management [22]. Future research comparing AS-OCT and UBM in ocular surface neoplasia and pigmented lesions could clarify the strengths and limitations of each method, potentially leading to an integrated imaging approach that enhances diagnostic precision and clinical decision-making.

In conclusion, high-resolution swept-source OCT provided valuable information of the structure, topographic location, depth and lateral extent of an ocular surface lesion and was helpful in assisting the diagnosis. Real-time imaging of premalignant and malignant masses is expected to help exploring the pathophysiology of ocular surface tumors allowing earlier identification and prompt treatment [23]. The ultimate aim of the noninvasive imaging modalities is to improve the diagnostics of these disorders eventually without surgical excision which might decrease the need for invasive medical interventions.

## Data Availability

The data that support the findings of this study are available from the corresponding author upon reasonable request.

## References

[1] Choma M, Sarunic M, Yang C, Izatt J. Sensitivity advantage of swept source and fourier domain optical coherence tomography. Opt Express. 2003;11(18):2183–9. 10.1364/oe.11.002183. 19466106 10.1364/oe.11.002183

[2] Fujimoto JG, Brezinski ME, Tearney GJ, Boppart SA, Bouma B, Hee MR, Southern JF, Swanson EA. Optical biopsy and imaging using optical coherence tomography. Nat Med. 1995;1(9):970–2. 10.1038/nm0995-970. 7585229 10.1038/nm0995-970

[3] Wirbelauer C, Winkler J, Bastian GO, Häberle H, Pham DT. Histopathological correlation of corneal diseases with optical coherence tomography. Graefes Arch Clin Exp Ophthalmol. 2002;240(9):727–34. 10.1007/s00417-002-0518-3. 12271369 10.1007/s00417-002-0518-3

[4] Aboumourad RJ, Galor A, Karp CL. Case series: High-resolution optical coherence tomography as an optical biopsy in ocular surface squamous neoplasia. Optom Vis Sci. 2021;98(5):450–5. 10.1097/OPX.0000000000001684. 33967253 10.1097/OPX.0000000000001684 PMC13452047

[5] Bille JF, editor. High [internet]esolution imaging in microscopy and ophthalmology: [internet]ew frontiers in biomedical optics [Internet]. Cham (CH): Springer; 2019. 32091677

[6] Alvarez OP, Galor A, AlBayyat G, Karp CL. Update on imaging modalities for ocular surface pathologies. Curr Ophthalmol Rep. 2021;9(2):39–47. 10.1007/s40135-021-00265-1. 36093383 10.1007/s40135-021-00265-1 PMC9455836

[7] Venkateswaran N, Sripawadkul W, Karp CL. The role of imaging technologies for ocular surface tumors. Curr Opin Ophthalmol. 2021;32(4):369–378. doi:.10.1097/ICU.0000000000000771. 10.1097/ICU.0000000000000771 PMC9148215 33989235

[8] Venkateswaran N, Mercado C, Wall SC, Galor A, Wang J, Karp CL. High resolution anterior segment optical coherence tomography of ocular surface lesions: A review and handbook. Expert Rev Ophthalmol. 2021;16(2):81–95. 10.1080/17469899.2021.1851598. 36313187 10.1080/17469899.2021.1851598 PMC9611086

[9] Venkateswaran N, Galor A, Wang J, Karp CL. Optical coherence tomography for ocular surface and corneal diseases: a review. Eye Vis (Lond). 2018;5:13. 10.1186/s40662-018-0107-0. 29942817 10.1186/s40662-018-0107-0 PMC5996489

[10] Shousha MA, Karp CL, Canto AP, Hodson K, Oellers P, Kao AA, Bielory B, Matthews J, Dubovy SR, Perez VL, Wang J. Diagnosis of ocular surface lesions using ultra-high-resolution optical coherence tomography. Ophthalmology. 2013;120(5):883–91. 10.1016/j.ophtha.2012.10.025. 23347984 10.1016/j.ophtha.2012.10.025 PMC3638067

[11] Szalai E, Németh G, Hassan Z, Módis L Jr. Noncontact evaluation of corneal grafts: Swept-Source fourier domain OCT versus High-Resolution Scheimpflug imaging. Cornea. 2017;36(4):434–9. 10.1097/ICO.0000000000001133. 28079690 10.1097/ICO.0000000000001133

[12] Ong SS, Vora GK, Gupta PK. Anterior segment imaging in ocular surface squamous neoplasia. J Ophthalmol. 2016;2016:5435092. 10.1155/2016/5435092. 27800176 10.1155/2016/5435092 PMC5069377

[13] Sripawadkul W, Khzam RA, Tang V, Zein M, Dubovy SR, Galor A, Karp CL. Anterior segment optical coherence tomography characteristics of conjunctival papilloma as compared to papilliform ocular surface squamous neoplasia. Eye (Lond). 2023;37(5):995–1001. 10.1038/s41433-022-02309-7. 36402855 10.1038/s41433-022-02309-7 PMC10050070

[14] Wylegala A, Sripawadkul W, Zein M, Alvarez OP, Al Bayyat G, Galor A, Karp CL. Topical 1% 5-fluorouracil eye drops as primary treatment for ocular surface squamous neoplasia: Long-term follow-up study. Ocul Surf. 2023;27:67–74. 10.1016/j.jtos.2022.12.002. 36476665 10.1016/j.jtos.2022.12.002

[15] Stevens SM, Reyes-Capo DP, Patel U, Choudhary A, Khzam RA, Tang V, Galor A, Karp CL, Dubovy S. Clinical and optical coherence tomography comparison between ocular surface squamous neoplasia and squamous metaplasia. Cornea. 2023;42(4):429–34. 10.1097/ICO.0000000000003039. 35439777 10.1097/ICO.0000000000003039 PMC9547982

[16] Atallah M, Joag M, Galor A, Amescua G, Nanji A, Wang J, Perez VL, Dubovy S, Karp CL. Role of high resolution optical coherence tomography in diagnosing ocular surface squamous neoplasia with coexisting ocular surface diseases. Ocul Surf. 2017;4688–95. 10.1016/j.jtos.2017.03.003. 10.1016/j.jtos.2017.03.003 PMC5610925 28347855

[17] Nanji AA, Sayyad FE, Galor A, Dubovy S, Karp CL. High-Resolution optical coherence tomography as an adjunctive tool in the diagnosis of corneal and conjunctival pathology. Ocul Surf. 2015;13(3):226–35. 10.1016/j.jtos.2015.02.001. 26045235 10.1016/j.jtos.2015.02.001 PMC4498969

[18] Theotoka D, Wall S, Galor A, Sripawadkul W, Khzam RA, Tang V, Sander DL, Karp CL. The use of high resolution optical coherence tomography (HR-OCT) in the diagnosis of ocular surface masqueraders. Ocul Surf. 2022;24:74–82. 10.1016/j.jtos.2022.02.003. 35231640 10.1016/j.jtos.2022.02.003 PMC9058205

[19] Vempuluru VS, Jakati S, Godbole A, Mishra DK, Mohamed A, Kaliki S. Spectrum of AS-OCT features of ocular surface tumors and correlation of clinico-tomographic features with histopathology: a study of 70 lesions. Int Ophthalmol. 2021;41(11):3571–86. 10.1007/s10792-021-01939-2. 34241759 10.1007/s10792-021-01939-2

[20] Vizvári E, Skribek Á, Polgár N, Vörös A, Sziklai P, Tóth-Molnár E. Conjunctival melanocytic Naevus: diagnostic value of anterior segment optical coherence tomography and ultrasound biomicroscopy. PLoS ONE. 2018;13(2):e0192908. 10.1371/journal.pone.0192908. 29444155 10.1371/journal.pone.0192908 PMC5812659

[21] Shields CL, Fasiuddin AF, Mashayekhi A, Shields JA. Conjunctival Nevi: clinical features and natural course in 410 consecutive patients. Arch Ophthalmol. 2004;122(2):167–75. 10.1001/archopht.122.2.167. 14769591 10.1001/archopht.122.2.167

[22] Zhou SY, Wang CX, Cai XY, Huang D, Liu YZ. Optical coherence tomography and ultrasound biomicroscopy imaging of opaque Corneas. Cornea. 2013;32(4):e25–30. 10.1097/ICO.0b013e318261eb2b. 23073488 10.1097/ICO.0b013e318261eb2b PMC3993526

[23] Monroy D, Serrano A, Galor A, Karp CL. Medical treatment for ocular surface squamous neoplasia. Eye (Lond). 2023;37(5):885–93. 10.1038/s41433-023-02434-x. 36754986 10.1038/s41433-023-02434-x PMC10050251

